# Resiliency, Stress, and Culture Shock: Findings from a Global Health Service Partnership Educator Cohort

**DOI:** 10.5334/aogh.3387

**Published:** 2021-11-30

**Authors:** Kiran Mitha, Sadath Ali Sayeed, Maria Lopez

**Affiliations:** 1 Seed Global Health, Boston, MA, US; 2 David Geffen School of Medicine at UCLA, Los Angeles, CA, US; 3 Harvard Medical School, Cambridge, MA, US; 4 Formerly affiliated with Seed Global Health, US

## Abstract

**Background:**

Global health field assignments for medical and nursing professionals include a wide variety of opportunities. Many placements often involve individuals practicing in settings very different from their home environments, relying on their professional experience to help bridge cultural and clinical divides.

**Objectives:**

There is limited information about the individual factors that might lead to successful longer-term global health experiences in non-disaster settings. In this paper, we report on one cohort of health professionals’ experiences of culture shock, stress, and resiliency as volunteers within the Global Health Service Partnership (GHSP), a public-private collaboration between Seed Global Health, the US Peace Corps, and the US Presidents Plan for Emergency Aids Relief (PEPFAR) that placed American medical and nursing educators in five African countries facing a shortage of health professionals.

**Methods:**

Using the tools of Project PRIME (Psychosocial Response to International Medical Electives) as a basis, we created the GHSP Educator Support Survey to measure resiliency, stress, and culture shock levels in a cohort of GHSP volunteers during their year of service.

**Findings:**

In our sample, participants were likely to experience lower levels of resiliency during initial quarters of global health placements compared to later timepoints. However, they were likely to experience similar stress and culture shock levels across quarters. Levels of preparedness and resources available, and medical needs in the community where the volunteer was placed played a role in the levels of resiliency, stress, and culture shock reported throughout the year.

**Conclusion:**

The GHSP Educator Support Survey represented a novel attempt to evaluate the longitudinal mental well-being of medical and nursing volunteers engaged in intense, long-term global health placements in high acuity, low resource clinical and teaching settings. Our findings highlight the need for additional research in this critical area of global health.

## Introduction

Global health field assignments for medical and nursing professionals include a wide variety of opportunities. Many placements often involve individuals practicing in settings very different from their home environments, relying on their professional knowledge to help naturally bridge cultural and clinical divides. Academic institutions and non-governmental organizations have increasingly invested resources in preparing healthcare professionals for the demands of working internationally, yet many of these resources are geared toward trainees in short-term immersion experiences, rather than professionals embedded in local communities for prolonged periods of time [1,2,3,4,5,6]. A robust body of literature has previously described the negative mental health outcomes of individuals working long-term in disaster relief settings such as PTSD, depression, and anxiety [7,8,9]. Few studies, however, have evaluated individual factors that might lead to successful long-term global health experiences in non-disaster settings.

Culture shock is a term often used to encompass the feelings of anxiety or discomfort a person experiences in an unfamiliar social environment [10,11,12]. The “stage theory” of culture shock includes a five-stage model: honeymoon, frustration, adjustment, acceptance, and reentry. A study of culture shock in social workers placed within rural communities in Canada determined the temporal progression through the five stages and demonstrated a curvilinear relationship between lack of well-being (culture shock) and time, with the lowest well-being at month 6 of placement and return to baseline well-being at month 12 [13]. While culture shock is commonly reported during global health placements [13,14,15,16,17,18,19], this curvilinear relationship has not been documented in long-term medical volunteers.

In addition to stress from cultural unfamiliarity, healthcare providers experience work-related stress that can significantly impact their well-being. Stress may lead to negative clinical consequences, such as medical errors, compassion fatigue, and unprofessionalism [20]. Stress can also lead to negative personal consequences, such as chronic fatigue, substance abuse, mental distress, and suicidal ideation [21,22,23,24]. This can be particularly heightened in settings where individuals are physically separated from their usual sources of support – friends, family, and community in their home countries.

A growing body of literature has evaluated the ability of resilience to counterbalance stress and burnout among healthcare workers. Resilience refers to an individual’s ability to overcome adversity and is a multifactorial construct that varies based on characteristics such as age, gender, time, and context [25,26,27]. Two areas previously identified as particularly challenging for healthcare workers are working in areas of resource deprivation and working in remote or rural areas, both of which apply to many settings where international healthcare workers are deployed [28]. Importantly, resilience is not a static internal quality but can be improved or worsened by environment. Studies of disaster relief workers have shown that contextual factors that improve resiliency may include pre-departure training, team-building efforts, in-country support and recognition, and formal re-entry assistance [29].

In this paper, we report on one cohort of health professionals’ experiences of culture shock, stress, and resiliency as volunteers within the Global Health Service Partnership (GHSP). GHSP was a public-private collaboration between Seed Global Health, the US Peace Corps, and the US Presidents Plan for Emergency Aids Relief (PEPFAR) that placed American medical and nursing educators in five African countries facing a shortage of health professionals between 2013–2018. Characteristics of the Global Health Service Partnership (GHSP) program included immersion into moderate to high acuity clinical settings, responsibilities of caring for a high volume of patients while providing trainee education, language differences, frequent limitations in available medical and human resources, diagnostic unfamiliarity with local diseases, and different hierarchical structures for clinical personnel.

## Methods

During the initial years of the GHSP program, multiple survey tools were developed to understand areas of needed programmatic support and quality improvement for the unique circumstances of practicing medical and nursing educators. In 2016, we aimed to combine these tools with validated questionnaires to specifically assess resiliency, stress, and culture shock. Based on pilot survey data and internal consensus, we chose to evaluate individual pre-departure preparation, previous clinical and teaching experience in both underserved domestic and international settings, familial circumstances, and resource variability across placements as unique factors of the GHSP experience.

Concurrently, an ongoing multi-institutional study called Project PRIME (Psychosocial Response to International Medical Electives) was developed by the Midwest Consortium of Global Child Health Educators in 2015 to evaluate medical trainee experiences during short-term global health electives around readiness, stress, and culture shock [30,31,32]. It included previously validated tools—the Connor-Davidson Resilience Scale (CD-RISC 10) to measure resiliency, the Perceived Stress Scale (PSS) to measure stress, and a modified version of the “Culture Shock Profile” questionnaire to evaluate culture shock. With permission, we adapted the validated tools from the PRIME protocol with our existing GHSP questionnaires and formulated the GHSP Educator Support Survey.

**Figure 1:**
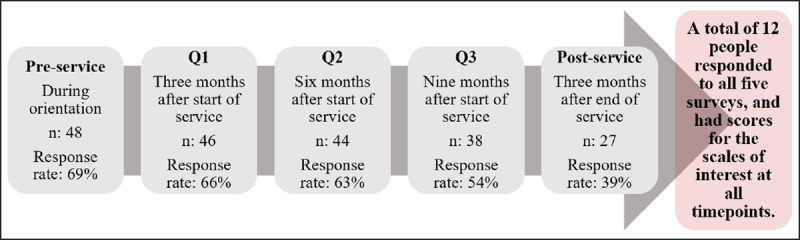
Survey timeline and participant responses.

In July 2016, 70 educators were deployed to partner sites in Liberia, Malawi, Swaziland, Tanzania, or Uganda for a period of one year and all were initially invited to participate in this study. The GHSP Educator Support Survey consisted of five surveys shared with GHSP educators before, during, and after their service. The first survey (pre-service) was administered to educators during their orientation week in Washington DC, with the following three surveys (Q1, Q2, Q3) administered quarterly to volunteers during their in-country placements. The final survey (post-service) was administered 3 months after completing their service (***Figure 1***). Quarterly surveys were selected in order to capture multiple points in the year-long placement and progression through the stages of culture shock without putting undue survey burden on GHSP educators. Surveys were administered electronically, and all data was de-identified. Quarterly response rates varied from 54–69%, with the exception of the post-service survey, which had a response rate of 39% (***Figure 1***). Data was cleaned and analyzed using SPSS.

Although frequencies and means are reported for all survey responses, statistical analyses were conducted only for individuals who completed all five surveys (n = 12) and who had scores for the Resiliency, Stress, and Culture Shock Profile scales for all timepoints.

### Sample demographics and professional characteristics

The majority of respondents to all five surveys were nurses (67%), similar to the breakdown between nurse and physician educators in the overall GHSP cohort. In addition, half of respondents were married or partnered volunteers (50%), with the majority of spouses/partners accompanying educators to their country of service (67%). Members of the sample group were more likely to have children above the age of 18 (58%), with 8% of respondents with children under 18, and 33% of respondents without children. Most were not immigrants or children of immigrants to the US and spoke only one language. The majority had not previously visited their country of placement and did not know the local language spoken at their sites (***Table 1***).

**Table 1:** Key sample and overall cohort demographics.


FREQUENCIES		SAMPLE (N = 12)	ALL RESPONDENTS (N = 48)

Program	Nursing	67%	52%

	Physician	33%	48%

Relationship status	Married/partnered	50%	33%

	Single	33%	60%

	Widower/other	17%	6%

Will your significant other be accompanying you? *(for those who said they were married or partnered when asked about their relationship status)*	No, significant other will not be present during the trip	33%*	25%^

	Yes, significant other will be present during the trip	67%*	75%^

Do you have any children or dependents?	Yes, over 18 years old	58%	33%

	No	33%	63%

	Yes, under 18 years old	8%	4%

Have you previously traveled to the country or site of your placement?	Yes, my country, but not my site	8%	23%

	Yes, my site	0%	4%

	Neither	92%	73%

Were you or your parents born outside of the United States?	No	75%	81%

	Yes	25%	19%

In how many languages are you conversationally/medically fluent?	Just English	73%	59%**

	Two languages	18%	33%**

	Three languages	0%	4%**

	Four or more languages	9%	4%**

In your last position before joining GHSP, were you responsible for clinical and/or classroom teaching?	Not responsible for teaching	25%	21%

	Yes – clinical	42%	38%

	Yes – classroom	8%	13%

	Yes – clinical and classroom	25%	29%

In your last position before joining GHSP, were you working clinically?	Yes – outpatient	33%	21%

	Yes – inpatient	25%	25%

	Yes – in/out combined	25%	40%

	Yes – emergency	8%	10%

	Not working clinically	8%	13%

Prior to joining GHSP, did you participate in any of the following activities?	Domestic health disparities research	0%	13%

	International research	33%	26%

	International health advocacy	25%	31%

	International clinical work	25%	54%

	Stateside global health education	25%	39%

	International health education	42%	50%

	Domestic advocacy work	50%	52%

	Domestic clinical work in low resource settings	83%	78%

	Domestic health education	83%	87%


* *n* = 6. ^ *n* = 16. ** *n* = 46.

In their last position before becoming a GHSP educator, the majority of participants (58%) worked in high acuity settings (inpatient or emergency) and were responsible for clinical and/or classroom teaching (75%). Most participants had previous experience working in low-resource settings domestically (83%), although a smaller percentage had clinical experience in an international setting (25%) (***Table 1***).

## Results

### Resiliency

The Connor-Davidson Resilience Scale (CD-RISC 10) consisted of a series of ten statements measuring agreement on areas such as ability to adapt to changes, coping with stress, staying focused and being able to handle life’s difficulties. Possible agreement options range from 0 (Not true at all) to 3 (Often true). Answers were then summed to generate a resiliency score, with possible scores ranging from 0 to 30 points. Participants with higher scores are said to have higher resiliency. Participant’s resiliency levels were assessed across all five surveys.

**Figure 2:**
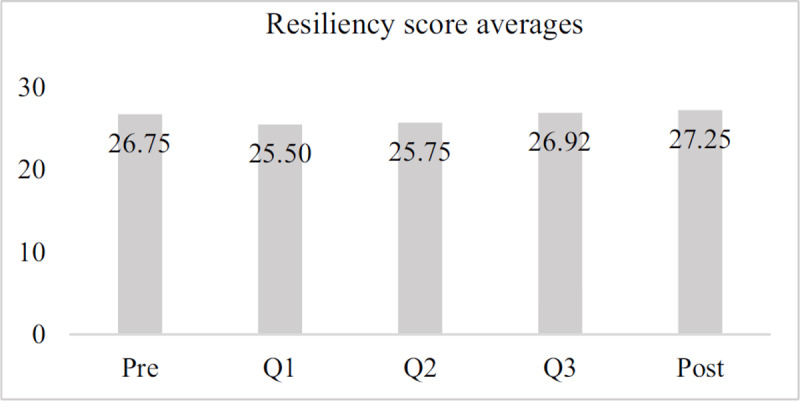
Average resiliency scores across time, sample group.

For the subset of participants who completed all five surveys, resiliency score averages stayed mostly the same during service, dipping slightly in Q1, but increasing steadily thereafter, with the highest resiliency scores being reported during the post-test (***Figure 2***).

**Figure 3:**
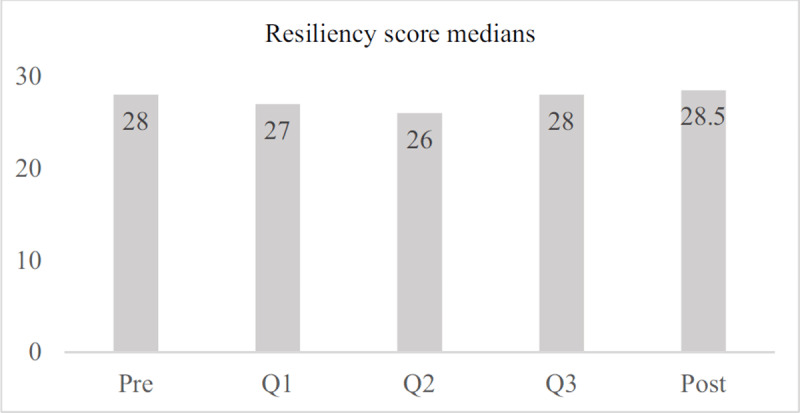
Resiliency score medians across time, sample group.

For this group, however, there were statistically significant differences across medians for resiliency (***Figure 3***). Resiliency scores in Q1 (Mdn = 27) were statistically significantly lower than in Q3 (Mdn = 28, *Z* = –2.063, p = 0.039) and in the post-departure timepoint (Mdn = 28.5, *Z* = –2.541, p = 0.011). In addition, Q2 Resiliency scores (Mdn = 26) were statistically significantly lower than Post Resiliency scores (Mdn = 28) (*Z* = –2.162, p = 0.031).

### Stress

The Perceived Stress Scale (PSS) consisted of a series of ten questions, assessing how often participants have felt able to cope effectively with stress in the recent past. Agreement options range from 0 (Never) to 4 (Very often). Answers are then summed to generate a stress score, with possible scores ranging from 0 to 40, with higher scores indicating higher stress levels. Participant’s stress levels were assessed across all five surveys.

**Figure 4:**
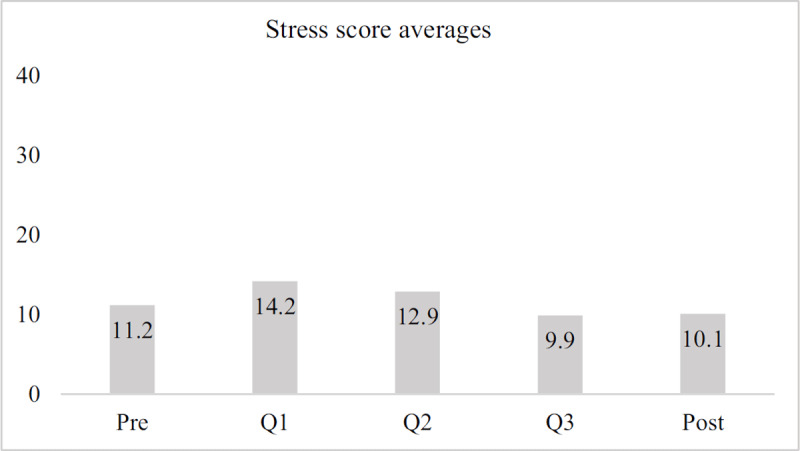
Average stress scores across time, sample group.

Overall, the average stress score across timepoints for the subset of participants who completed all five surveys was 11.66 out of 40. Stress levels for GHSP educators peaked in Q1, and decreased to below average levels in both Q3 and post-service (***Figure 4***).

For this group, no statistically significant differences across assessment time points were found.

### Culture shock

The Culture Shock Profile Questionnaire measured the intensity with which participants experienced a series of 33 positive and negative feelings. The intensity of the feeling was measured from 0 (None) to 3 (Great). Answers were then summed to generate a culture shock score, with possible scores ranging from 0 to 99. The higher the scores, the more culture shock the participants experienced. Culture shock was measured every quarter once participants were in their placement (Q1–Q3) and once participants finished their service and left their service site (post) to assess for reverse culture shock.

**Figure 5:**
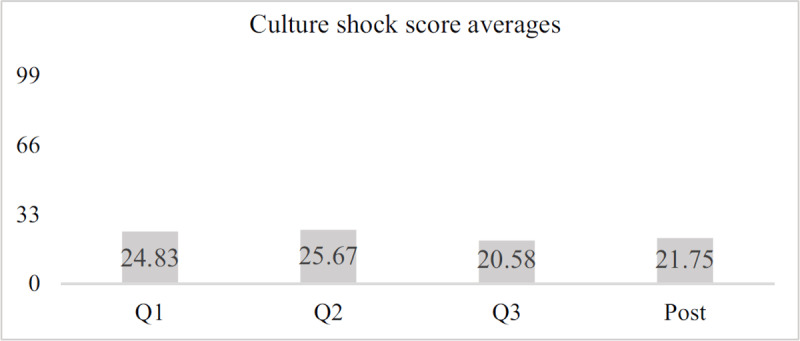
Average culture shock scores across time, sample group.

Overall, culture shock score averages for the subset of participants who completed all five surveys were highest in Q2, decreasing in Q3 and increasing slightly again during the post-service survey (***Figure 5***).

For this group, however, no statistically significant differences were found across the assessment time points.

On sub-analysis, significant differences in the levels of culture shock were found by participants during Q1, based on self-reported levels of feelings of preparedness for their teaching role during their GHSP year during the pre-test survey (χ2(2) = 6.317, p = 0.042). The median Q1 culture shock score for the group that reported feeling somewhat prepared was 27, while the median culture shock score for the group that reported feeling very prepared was 11.

### Associations

Correlation analyses were conducted using data from the subset of participants who completed all five surveys to determine if there were any significant associations between educator characteristics and other variables such as resiliency and culture shock scores.

Self-reported levels of preparedness at the predeparture timepoint correlated with resiliency in multiple quarters (Q2 and Q3). Low levels of preparedness at the predeparture timepoint correlated with high levels of culture shock throughout the year and even after their return to the US. Respondents who felt overwhelmed by the medical needs in their community reported higher levels of stress and culture shock across quarters (Q1 and Q3) as well as lower levels of resiliency (Q1, Q2, post). Educators with sufficient teaching resources felt higher levels of stress to provide adequate education to their students across quarters (Q1–Q3) compared to educators without sufficient teaching resources. Higher resiliency levels across the year correlated with respondents feeling fully reintegrated in their home culture on their return to the US (***Table 2***).

**Table 2:** Correlation analyses results, sample group.


SPEARMAN CORRELATIONS		CORRELATION COEFFICIENT	SIG. (2-TAILED)	N

Pre – At this time, how prepared do you feel: For your teaching role during your GHSP year?	Culture shock Q1	–.643*	0.024	12

	Culture shock Q3	–.610*	0.035	12

	Culture shock Post	–.652*	0.022	12

Q1 – I feel overwhelmed by the medical needs in this community.	Culture shock Q1	.744**	0.005	12

	Culture shock Q3	.829**	0.001	12

	Resiliency Q1	–.704*	0.011	12

	Resiliency Q2	–.644*	0.024	12

	Resiliency Post	–.592*	0.043	12

	Stress Q1	.703*	0.011	12

	Stress Q3	.679*	0.015	12

Q3 – I feel overwhelmed by the medical needs in this community.	Stress Q1	.658*	0.02	12

	Culture shock Q1	.657*	0.02	12

	Culture shock Q2	.611*	0.035	12

	Culture shock Post	.678*	0.015	12

Q1 – There are sufficient resources to provide adequate education to my students.	Stress Q3	.679*	0.015	12

Q2 – There are sufficient resources to provide adequate education to my students.	Stress Q1	.587*	0.045	12

	Stress Q3	.704*	0.011	12

Q3 – There are sufficient resources to provide adequate education to my students.	Stress Q2	.710*	0.014	11

Post – I am fully reintegrated in my home culture.	Resiliency Q1	.804**	0.009	9

	Resiliency Q2	.814**	0.008	9

	Resiliency Q3	.785*	0.012	9

	Resiliency Post	.845**	0.004	9

Respondent is single	Q1 – In the last two weeks, how responsible did you feel for your patients’ clinical outcomes on average?	–.798*	0.032	7

	Q3 – In the last two weeks, how responsible did you feel for your patients’ clinical outcomes on average?	–.828*	0.042	6

	Stress Q1	–.590*	0.043	12

	Stress Post	–.670*	0.017	12


## Discussion

The GHSP Educator Support Survey represented a novel attempt to evaluate the longitudinal mental well-being of medical and nursing volunteers engaged in intense, long-term global health placements in high acuity, low resource clinical and teaching settings. Given the competing professional demands of work and challenges around internet connectivity, our response rate for completing all 5 surveys was low (12 out of 70 participants). Thus, our results must be interpreted with caution. Those educators who may have been experiencing higher levels of stress or culture shock may have been less likely to respond to all 5 surveys, leading to disproportionately positive results in our response sample.

In our sample, participants were likely to experience similar resiliency, stress, and culture shock levels across quarters. This may be due to the limited sample size of the respondents, where average scores may not be reflective of individual variations. The median resiliency scores did decrease during the initial quarters of service, likely reflecting the period of adjustment of individuals to their new professional roles and environment. There may also be robustness of these parameters in the global health volunteer population, which may indicate a potential value of using these tools to assess baseline levels of stress and resiliency as part of the selection process for global health placements. Respondents in our sample had overall high levels of resiliency across timepoints (average 26.4 out of 30), low levels of stress (average 11.66 out of 40), and low levels of culture shock (23.2 out of 99).

Another possible signal from our study suggests the value of adequate self-preparation prior to embarking on long-term global health placements. Resiliency positively correlated to self-reported level of preparedness at the pre-service survey timepoint. Self-preparation was, in addition to the formal 1-month orientation included as part of the GHSP program. This suggests that those individuals with high levels of self-motivation to engage in self-study may fare better during their global health deployments.

Respondents who felt overwhelmed by the medical needs in their community reported higher levels of stress and culture shock across quarters as well as lower levels of resiliency. Although global health placements often prioritize settings with high medical need, additional research needs to be done to determine what factors in the medical setting may specifically impact resiliency. Interestingly, those individuals with sufficient teaching resources also reported increased stress to provide medical education, potentially implying that in settings where educational resources have been prioritized, there is added pressure for educators to perform at a high level.

Lastly, global health programs may benefit from an intentional process for professional reintegration on the completion of deployment, particularly for those individuals who struggle during their placement. Higher resiliency levels across the year correlated with respondents feeling fully reintegrated in their home culture on their return to the US, potentially implying that those with lower resiliency did not reintegrate fully on return to the US.

## Conclusion

Our findings highlight the need for further structured study on how global health experiences impact the mental well-being of medical and nursing professionals. Future efforts should also be directed toward better understanding factors that best support healthcare workers in settings that can be anticipated to generate culture shock, stress, and test professional and personal resiliency.

## Additional Files

The additional files for this article can be found as follows:

10.5334/aogh.3387.s1 Appendix 1. Table 3. Additional statistically significant Pearson correlations.

10.5334/aogh.3387.s2 Appendix 2. Survey tools.
