# Supplementary material for: Resiliency, Stress, and Culture Shock: Findings from a Global Health Service Partnership Educator Cohort
Source: Ann Glob Health. 2021 Nov 30;87(1):120. doi: 10.5334/aogh.3387 (PMC8641533; doi:10.5334/aogh.3387)
Supplement: Appendix 1. — Table 3. Additional statistically significant Pearson correlations. [file agh-87-1-3387-s1.pdf]

Appendix

Table 3. Additional statistically significant Pearson correlations.

Pearson Correlations		Correlation coefficient	Sig. (2-tailed)	N
Resiliency Pre	Resiliency Q1	.686*	0.014	12
	Resiliency Q2	.857**	0.000	12
Resiliency Q1	Resiliency Q2	.770**	0.003	12
Resiliency Q3	Resiliency Pre	.636*	0.026	12
	Resiliency Q1	.817**	0.001	12
	Resiliency Q2	.670*	0.017	12
Resiliency Post	Resiliency Pre	.623*	0.031	12
	Resiliency Q1	.905**	0.000	12
	Resiliency Q2	.757**	0.004	12
	Resiliency Q3	.885**	0.000	12
Stress Q1	Resiliency Post	-.707*	0.010	12
	Resiliency Q1	-.804**	0.002	12
	Resiliency Q2	-.578*	0.049	12
	Resiliency Q3	-.820**	0.001	12
Stress Q2	Resiliency Post	-.622*	0.031	12
	Resiliency Q1	-.647*	0.023	12
	Resiliency Q2	-.594*	0.042	12
	Resiliency Q3	-.664*	0.019	12
	Stress Q1	.582*	0.047	12
Stress Q3	Resiliency Pre	-.675*	0.016	12
	Resiliency Q3	-.638*	0.026	12
	Stress Q2	.618*	0.032	12
Stress Post	Resiliency Q3	-.666*	0.018	12
	Stress Q1	.595*	0.041	12
	Stress Q2	.727**	0.007	12
	Stress Q3	.672*	0.017	12
Culture shock Q1	Resiliency Post	-.698*	0.012	12
	Resiliency Pre	-.597*	0.040	12
	Resiliency Q1	-.848**	0.000	12
	Resiliency Q2	-.694*	0.012	12
	Resiliency Q3	-.761**	0.004	12
	Stress Q1	.863**	0.000	12
	Stress Q2	.589*	0.044	12
Culture shock Q2	Culture shock Q1	.722**	0.008	12
	Resiliency Q1	-.587*	0.045	12
	Resiliency Q2	-.599*	0.040	12
	Resiliency Q3	-.706*	0.010	12
	Stress Post	.586*	0.045	12
	Stress Q1	.689*	0.013	12
	Stress Q2	.856**	0.000	12
Culture shock Q3	Resiliency Pre	-.715**	0.009	12
	Resiliency Q1	-.595*	0.041	12
	Resiliency Q2	-.762**	0.004	12

	Resiliency Q3	-.595*	0.041	12
	Stress Post	.590*	0.044	12
	Stress Q1	.682*	0.015	12
	Stress Q3	.598*	0.040	12
Culture shock Post	Culture shock Q1	.588*	0.044	12
	Culture shock Q2	.734**	0.007	12
	Culture shock Q3	.691*	0.013	12
	Resiliency Post	-.718**	0.009	12
	Resiliency Q1	-.616*	0.033	12
	Resiliency Q3	-.911**	0.000	12
	Stress Post	.826**	0.001	12
	Stress Q1	.778**	0.003	12
	Stress Q2	.666*	0.018	12
	Stress Q3	.756**	0.004	12

Table 4. Additional statistically significant Spearman correlations.

Spearman Correlations		Correlation Coefficient	Sig. (2-tailed)	N
Pre - Was responsible for clinical and/or classroom teaching in their last position before GHSP.	Pre - At this time, how prepared do you feel: To live in a new environment during your GHSP year?	.579*	0.048	12
Pre - At this time, how prepared do you feel: For the clinical work you will be doing during your GHSP year?	Pre - At this time, how prepared do you feel: For your GHSP year overall?	.706*	0.01	12
	Pre - At this time, how prepared do you feel: For your teaching role during your GHSP year?	.739**	0.006	12
Q1 - At this moment, I would like to incorporate global health into my career when I finish GHSP.	Stress Pre	-.825**	0.006	9
	Culture shock Q2	.822**	0.007	9
	Culture shock Post	.688*	0.041	9
Q1 - I am part of a strong community in my site that includes host nationals [for example, Church, exercise group, Rotary Club]	Stress Q3	-.598*	0.04	12
Q1 - I am part of a strong GHSP community at my site/city	Resiliency Post	.593*	0.042	12
	Stress Q3	-.815**	0.001	12
Q1 - I can communicate easily with my counterpart	Q1 - My GHSP counterpart here is helpful	.621*	0.031	12
	Resiliency Q1	.586*	0.045	12

Q1 - I feel that I have the ability to innovate in my position.	Resiliency Pre	.591*	0.043	12
Q1 - I have a clear sense of how I can be helpful.	Q1 - I have a clear sense of my role.	.903**	0.0000	12
Q1 - Language is a challenge when communicating with my colleagues	Resiliency Post	-.672*	0.017	12
Q1 - My colleagues in the clinical setting are helpful	Q1 - I can communicate easily with my counterpart	.777**	0.003	12
	Q1 - My GHSP counterpart here is helpful	.687*	0.014	12
Q2 - At this moment, I would like to incorporate global health into my career when I finish GHSP.	Culture shock Post	.688*	0.041	9
	Culture shock Q2	.822**	0.007	9
	Q2 - I have a clear sense of how I can be helpful.	-.732*	0.025	9
	Stress Pre	-.825**	0.006	9
Q2 - I am part of a strong community in my site that includes host nationals [for example, Church, exercise group, Rotary Club]	Stress Q2	-.669*	0.017	12
	Stress Q3	-.621*	0.031	12
Q2 - I am part of a strong expat community in my city	Q2 - There is a good support network here to help me deal with difficult situations	.604*	0.038	12
Q2 - I have a clear sense of how I can be helpful.	Culture shock Q2	-.648*	0.023	12
	Q2 - I have a clear sense of my role.	.899**	0.000	12
	Stress Pre	.665*	0.018	12
Q2 - I have been able to make a positive difference in my work.	Q2 - I feel that I have the ability to innovate in my position.	.746**	0.005	12
Q2 - Language is a challenge when communicating with my students/trainees	Resiliency Post	-.587*	0.045	12
	Resiliency Q3	-.615*	0.033	12
Q2 - My GHSP counterpart here is helpful	Q2 - I can communicate easily with my counterpart	.738**	0.006	12
Q3 - I am part of a strong community in my site that includes host nationals [for example, Church, exercise group, Rotary Club]	Q3 - I am part of a strong expat community in my city	-.847**	0.001	12
Q3 - I am part of a strong expat community in my city	Stress Q2	.661*	0.019	12
Q3 - I have a clear sense of my role.	Q3 - I have a clear sense of how I can be helpful.	.793**	0.002	12
Q3 - Language is a challenge when communicating with my	Q3 - Language is a challenge when communicating with	.844**	0.001	12

colleagues	my students/trainees			
Q3 - My GHSP counterpart here is helpful	Culture shock Q3	.768**	0.006	11
	Stress Q1	.670*	0.024	11
Q3 - There are sufficient resources to provide adequate care for my patients	Culture shock Q3	-.627*	0.029	12
Post - At this moment, I would like to incorporate global health into my career when I finish GHSP.	Post - I feel that I have the ability to innovate in my position.	.747**	0.008	11
	Post - Working in global health is a core part of my self-identity.	.873**	0.001	10
	Post - Working in global health is a core part of my self-identity.	.873**	0.001	10
Post - I am fully reintegrated in my home culture.	Culture shock Post	-.692*	0.039	9
	Culture shock Q1	-.748*	0.02	9
	Post - Prior to leaving my GHSP site, I felt overwhelmed by the process of returning home.	-.690*	0.04	9
	Post - Reintegration to my life in the U.S after GHSP was a challenge.	-.736*	0.024	9
	Stress Q1	-.751*	0.02	9
Post - I feel integrated at my site/job	Culture shock Q1	-.719**	0.008	12
	Post - There is a good support network here to help me deal with difficult situations	.817**	0.001	12
Post - I feel overwhelmed by the medical needs in this community.	Culture shock Post	.676*	0.022	11
	Stress Post	.607*	0.048	11
	Stress Q1	.644*	0.033	11
	Stress Q3	.819**	0.002	11
Post - I feel that I have the ability to innovate in my position.	Post - At this moment, I would like to incorporate global health into my career when I finish GHSP.	.747**	0.008	11
	Post - Working in global health is a core part of my self-identity.	.700*	0.024	10
	Post - Working in global health is a core part of my self-identity.	.700*	0.024	10
Post - I have a clear sense of how I can be helpful.	Culture shock Post	-.646*	0.023	12
	Post - I have a clear sense of my role.	.753**	0.007	11
	Stress Post	-.594*	0.042	12

Post - I have a clear sense of my role.	Post - I have a clear sense of how I can be helpful.	.753**	0.007	11
Post - Prior to leaving my GHSP site, I felt overwhelmed by the process of returning home.	Culture shock Post	.849**	0.004	9
	Culture shock Q1	.718*	0.029	9
	Culture shock Q3	.704*	0.034	9
	Post - I am fully reintegrated in my home culture.	-.690*	0.04	9
	Resiliency Post	-.693*	0.038	9
	Resiliency Q1	-.886**	0.001	9
	Resiliency Q3	-.913**	0.001	9
	Stress Q1	.863**	0.003	9
Post - Reflecting on my work with GHSP, I was able to make a positive difference at my site.	Post - Working in global health is a core part of my self-identity.	.767*	0.016	9
	Stress Pre	-.720*	0.029	9
Post - Reintegration to my life in the U.S after GHSP was a challenge.	Culture shock Post	.668*	0.049	9
	Culture shock Q2	.795*	0.01	9
	Post - I am fully reintegrated in my home culture.	-.736*	0.024	9
	Stress Q1	.674*	0.047	9
Post - The expectations of my skills (from staff, providers, and patients) match my level of training.	Post - I have been able to make a positive difference in my work.	.751**	0.008	11
Post - There is a good support network here to help me deal with difficult situations	Culture shock Q1	-.682*	0.015	12
	Resiliency Q2	.702*	0.011	12
Post - Working in global health is a core part of my self-identity.	Post - Reflecting on my work with GHSP, I was able to make a positive difference at my site.	.767*	0.016	9
Resiliency Q2	Pre - At this time, how prepared do you feel: To live in a new environment during your GHSP year?	.591*	0.043	12
Resiliency Q3	Has children or dependents	.627*	0.029	12
	Pre - At this time, how prepared do you feel: For your GHSP year overall?	.627*	0.029	12
	Significant other will be present in trip	-.853*	0.031	6
Stress Q1	Has children or dependents	-.625*	0.03	12
	Is single	-.590*	0.043	12
Stress Post	Is single	-.670*	0.017	12
Culture shock Q1	Has children or dependents	-.586*	0.045	12

Culture shock Post	Pre - At this time, how prepared do you feel: For your GHSP year overall?	-.608*	0.036	12
