# Supplementary material for: Resiliency, Stress, and Culture Shock: Findings from a Global Health Service Partnership Educator Cohort
Source: Ann Glob Health. 2021 Nov 30;87(1):120. doi: 10.5334/aogh.3387 (PMC8641533; doi:10.5334/aogh.3387)
Supplement: Appendix 2. — Survey tools. [file agh-87-1-3387-s2.pdf]

PRE-SERVICE SURVEY (ADMINISTERED AT DC ORIENTATION, T0)

Thank you for participating in this project. The data being collected in these surveys is meant to help the GHSP team (Seed and Peace Crops) understand the personal and contextual factors that influence your experience with GHSP, specifically looking at resiliency, level of stress, and the experience of culture shock. This will then be used to identify support methods to promote successful functioning before, during, after service.

Please take 30 minutes to complete the pre-departure survey today, prior to completing Orientation. Your MGH ID is being used in order to connect your answers with future survey responses, but will be replaced with a unique study number so no one analyzing the data can connect answers to a specific individual. Additionally, the data collected online will be deleted from the system once each survey round is complete. Thank you for your time!

Demographics

1. ID NUMBER

2. What is your relationship status?
   - Single
   - Married/partnered
   - Other (please specify)

3. Will your significant other be accompanying you during your GHSP placement?
   - Yes
   - No
   - Not applicable

4. Do you have any children or dependents? [select all that apply]
   - No
   - Yes – children/dependents under 18 years of age
   - Yes – children/dependents 18 years of age or older

5. How often do you plan to communicate with your support network back home? Daily/weekly/monthly/a few times a year

6. How do you plan to communicate with your support network back home? (check all that apply)
   - Calling (telephone or internet-based)
   - E-mails
   - Blogs
   - Facebook/Twitter (social media)
   - Texting/WhatsApp
   - Video calls (Skype/Facetime)
   - Postal letters
   - Other (please specify)
7. Were you or your parent(s) born outside the US?
   - I was
   - One or both of my parents were
   - Both my parents and I were
   - Neither

   If yes, what country/countries:

8. How many languages do you speak? (Conversationally and/or medically fluent)

Professional Experiences

9. In your last position before joining GHSP, were you responsible for clinical and/or classroom teaching?
    - Yes - clinical
    - Yes – classroom
    - Yes – Clinical and classroom
    - Not teaching

10. In your last position before joining GHSP, were you working clinically? [select all that apply]
    - Yes - Outpatient
    - Yes - Inpatient
    - Yes - Outpatient and inpatient combined
    - Yes - Emergency
    - Not working clinically

Global Exposure

11. Have you previously traveled to the country or site of your placement?
    - Yes – my country, but not site
    - Yes – my site
    - Neither

12. How fluent in the predominant language at your placement site are you currently?
    - Do not know the language at all
    - Know a little of the language
    - Conversationally fluent
    - Medically fluent
    - Conversationally and medically fluent

13. Please indicate the degree to which the following factors influenced your decision to participate in GHSP.

- To learn what it is like to practice medicine in a different cultural setting
- To practice medicine with resource limitations
- To improve my language fluency
- To gain an understanding of a different culture
- To improve my procedural skills
- To make a positive difference in the world
- To improve my knowledge of tropical medicine
- To improve my knowledge of health systems globally
- To help reduce health disparities
- To improve my diagnostic skills
- To have the opportunity to travel
- To pursue this sort of work due to faith-based motivations
- To work with a specific patient population
- To help train the next generation of health care workers

Not at all influential	Slightly influential	Somewhat influential	Very influential	Extremely influential

14. Are there other factors that strongly influenced your decision to participate in GHSP?

(free text)

15. Prior to joining GHSP, did you participate in any of the following activities? [Yes/no]

International research
International health advocacy
International health education (precepting, teaching, mentorship and/or training)
International clinical work
Stateside global health education
Domestic clinical work in low resource settings
Domestic advocacy work
Domestic health disparities research
Domestic health education (precepting, teaching, mentorship and/or training)

16. How likely are you to incorporate these components of global health into your career once you return?

	Very unlikely	Unlikely	Unsure	Somewhat likely	Very likely
International research					
International health advocacy					
International health education (precepting, teaching, mentorship and/or training)					
International clinical work					
Stateside global health education					
Domestic clinical work in low resource settings					
Domestic advocacy work					
Domestic health disparities research					
Domestic health education (precepting, teaching, mentorship and/or training)					

17. Please indicate which activities you did in anticipation of your GHSP placement **PRIOR** to DC Orientation: (Select all that apply)
    - Communication with members of the Seed Global Health team related to my **specific site placement and work responsibilities**
    - Communication with members of the Seed Global Health team related to **logistics** (packing, technology, etc.)
    - Communication with members of Peace Corps Post in my placement country
    - Communication with my counterpart/supervisor/another faculty member at my host site
    - Communication with previous or current GHSP educators
    - Communication with staff, current and incoming GHSP educators using the **discussion boards on the Seed Learning Community**
    - Participation in the Seed pre-departure course - "Strategies for Successful Teaching and Learning: Best Practices and Local Context" (includes participation in any or all sections)
    - Participation in simulation sessions pertaining to care of a patient in low resource settings (that you independently initiated)
    - Independent reading pertaining to providing clinical care
    - Independent reading pertaining to providing clinical teaching
    - Independent reading pertaining to providing classroom teaching

- Independent reading pertaining to the process of settling into a new culture (example: culture shock, resiliency, stress management, cultural competencies)
- Researching the country you will be placed (e.g., exploring culture, health system, and/or current affairs)
- Language study/course in the host language
- Independent travel to a low-resource country or region for recreation (within 6 months of orientation)
- Independent travel to a low-resource country or region for clinical work (within 6 months of orientation)

18. At this time, how prepared do you feel:
    - To live in a new environment during your GHSP year?
    - For the clinical work you will be doing during your GHSP year?
    - For your teaching role during your GHSP year?
    - For your GHSP year overall?

    Very unprepared
    Somewhat unprepared
    Somewhat prepared
    Very prepared
    N/A

    Please explain:

[CD-RISC 10 Resilience Assessment Tool[20,22,23]*]*

Please indicate how much you agree with the following statements as they apply to you over the last month, using the number scale below. If a particular situation has not occurred recently, answer according to how you think you may have felt.

	0=Not true at all	1=Rarely true	2= Sometimes true	3=Often true
19. I am able to adapt when changes occur				
20. I can deal with whatever comes my way				
21. I try to see the humorous side of things when I am faced with problems				
22. Having to cope with stress can make me stronger				
23. I tend to bounce back after illness, injury, or other hardships				
24. I believe I can achieve my goals, even if there are obstacles				
25. Under pressure, I stay focused and think clearly				
26. I am not easily discouraged by failure				
27. I think of myself as a strong person when dealing with life's challenges and difficulties				
28. I am able to handle unpleasant or painful feelings like sadness, fear, and anger				

[Perceived Stress Scale[27,28]]

29. In the past month, how often have you:

	Never	*Almost Never*	*Sometimes*	*Fairly Often*	*Very Often*
• Been upset because of something that happened unexpectedly?					
• Felt that you were unable to control the important things in your life?					
• Felt nervous and “stressed”?					
• Felt confident about your ability to handle your personal problems?					
• Felt that things were going your way?					
• Found that you could not cope with all the things that you had to do?					
• Been able to control irritations in your life?					
• Felt that you were on top of things?					
• Been angered because of things that were outside of your control?					
• Felt difficulties were piling up so high that you could not overcome them?					

Unpublished key for Perceived Stress Scale[27,28], not to be included in the survey:
Score assignment:
0=Never 1=Almost Never 2=Sometimes 3=Fairly Often 4=Very Often

----

30. What is the primary worry that you have at this time about your GHSP placement? (free text)

ON-SITE SURVEY, TO BE ADMINISTERED QUARTERLY DURING PLACEMENT

Thank you for participating in this project. The data being collected in these surveys is meant to help the GHSP team (Seed and Peace Crops) understand the personal and contextual factors that influence your experience with GHSP, specifically looking at resiliency, level of stress, and the experience of culture shock. This will then be used to identify support methods to promote successful functioning before, during, after service.

Please take 30 minutes to complete this survey. Your MGH ID is being used in order to connect your answers with future survey responses, but will be replaced with a unique study number so no one analyzing the data can connect answers to a specific individual. Additionally, the data collected online will be deleted from the system once each survey round is complete. Thank you for your time!

1. What personal or professional factors are impacting your well-being most at this point in time? (free-text)

[CD-RISC 10 Resilience Assessment Tool[20,22,23]]
Please indicate how much you agree with the following statements as they apply to you over the last month, using the number scale below. If a particular situation has not occurred recently, answer according to how you think you may have felt.

	0=Not true at all	1=Rarely true	2= Sometimes true	3=Often true
2. I am able to adapt when changes occur				
3. I can deal with whatever comes my way				
4. I try to see the humorous side of things when I am faced with problems				
5. Having to cope with stress can make me stronger				
6. I tend to bounce back after illness, injury, or other hardships				
7. I believe I can achieve my goals, even if there are obstacles				
8. Under pressure, I stay focused and think clearly				
9. I am not easily discouraged by failure				
10. I think of myself as a strong person when dealing with life's challenges and difficulties				
11. I am able to handle unpleasant or painful feelings like sadness, fear, and anger				

[Perceived Stress Scale[27,28]]

12. In the past month, how often have you:

	Never	*Almost Never*	*Sometimes*	*Fairly Often*	*Very Often*
• Been upset because of something that happened unexpectedly?					
• Felt that you were unable to control the important things in your life?					

• Felt nervous and "stressed"?					
• Felt confident about your ability to handle your personal problems?					
• Felt that things were going your way?					
• Found that you could not cope with all the things that you had to do?					
• Been able to control irritations in your life?					
• Felt that you were on top of things?					
• Been angered because of things that were outside of your control?					
• Felt difficulties were piling up so high that you could not overcome them?					

Unpublished key for Perceived Stress Scale[27,28], not to be included in the survey:
Score assignment:
0=Never 1=Almost Never 2=Sometimes 3=Fairly Often 4=Very Often

----

[Culture Shock Profile Questionnaire[14,26]]

Below is a list of 33 items that may or may not describe how you feel about your experiences in your new culture. Please indicate the intensity with which you are experiencing the feeling **today**.

FEELING	INTENSITY			
13. Enthusiastic	None	Slight	Moderate	Great
14. Impatient	None	Slight	Moderate	Great
15. A desire to leave the program	None	Slight	Moderate	Great
16. Happy	None	Slight	Moderate	Great
17. Energetic	None	Slight	Moderate	Great
18. Rejected	None	Slight	Moderate	Great
19. Purposeful	None	Slight	Moderate	Great
20. Pessimistic/hopeless	None	Slight	Moderate	Great
21. Contemptuous of local people	None	Slight	Moderate	Great
22. Angry	None	Slight	Moderate	Great
23. A need to complain	None	Slight	Moderate	Great
24. Creative	None	Slight	Moderate	Great
25. Confident	None	Slight	Moderate	Great
26. Ready to cry	None	Slight	Moderate	Great
27. Challenged	None	Slight	Moderate	Great
28. Cynical	None	Slight	Moderate	Great
29. A sense of discovery	None	Slight	Moderate	Great
30. Helpless	None	Slight	Moderate	Great
31. Optimistic	None	Slight	Moderate	Great
32. Inadequate	None	Slight	Moderate	Great

33. Isolated	None	Slight	Moderate	Great
34. Irritable	None	Slight	Moderate	Great
35. I need to "get out"	None	Slight	Moderate	Great
36. Disoriented	None	Slight	Moderate	Great
37. Excited	None	Slight	Moderate	Great
38. Accepted	None	Slight	Moderate	Great
39. A sense of loss	None	Slight	Moderate	Great
40. Overwhelmed	None	Slight	Moderate	Great
41. Fearful	None	Slight	Moderate	Great
42. Depressed	None	Slight	Moderate	Great
43. Frustrated	None	Slight	Moderate	Great
44. Exhausted	None	Slight	Moderate	Great
45. Apathetic	None	Slight	Moderate	Great

Unpublished key, not to be included in the survey:

0	1	2	3
None	*Slight*	*Moderate*	*Great*

Reverse the score for items 1, 4, 5, 7, 12, 13, 17, 19, 25, and 26 (if original score was a 0, reverse to 3; if original score was a 1, reverse to a 2; if original score was a 2, reverse to a 1; if original score was a 3, reverse to a 0). After reversing the scores for those 10 items, sum the entire 33 items. Scores must range from 0 to 99. (Theoretically, the higher the score, the more culture shock the trainee may be experiencing)

Training Conditions

	Strongly Disagree	Disagree	Neither Agree nor Disagre e	Agree	Strongly Agree	N/A
46. My GHSP counterpart here is helpful						
47. I can communicate easily with my counterpart						
48. My colleagues in the clinical setting are helpful						
49. My colleagues in the academic setting are helpful						
50. There is a good support network here to help me deal with difficult situations						
51. I am part of a strong GHSP community at my site/city						
52. I am part of a strong expat community in my city						
53. I am part of a strong community in my site that includes host nationals [for example, Church, exercise group, Rotary Club]						
54. Language is a challenge when communicating with my patients						
55. Language is a challenge when communicating with my colleagues						
56. Language is a challenge when communicating						

with my students/trainees						
57. I feel integrated at my site						
58. There are sufficient resources to provide adequate care for my patients						
59. There are sufficient resources to provide adequate education to my students						
60. I feel overwhelmed by the medical needs in this community.						
61. I have a clear sense of my role.						
62. I have a clear sense of how I can be helpful.						
63. I feel conflicted between my own ideas of medical care and a desire to respect local medical practices that differ.						
64. The expectations of my skills (from staff, providers, and patients) match my level of training.						
65. I have been able to make a positive difference in my work.						
66. I feel that I have the ability to innovate in my position.						
67. Working in global health is a core part of my self-identity.						

68. Please check the option that best describes your clinical role over the past 2 weeks:
    - Clinical provider with no bedside teaching
    - Clinical provider with some bedside teaching - patient care is the main activity, teaching is secondary
    - Clinical provider with equal responsibilities of patient care and teaching
    - Clinical provider with a lot of bedside teaching - teaching is the main activity, patient care is secondary
    - Clinical preceptor - no clinical responsibilities but support students with patients when needed
    - No clinical role in the past 2 weeks [skip to next section]

The following questions pertain to the acuity and clinical experience in your role.

69. Assess the average health status of the majority of your patients over the past 2 weeks:
    - Minimally sick (e.g. primarily outpatient issues, very few required hospitalization)
    - Moderately sick (at least half of the patients required hospitalization in a general inpatient ward)
    - Significantly sick (the majority of patients required hospitalization with frequent interventions)
    - Critically sick (the majority of patients required hospitalization and significant or life-saving interventions)
    - Not applicable (I did not care for patients over the past 5 days)

70. Estimate the percent of your patients that have died over the past 2 weeks (0 if none): _____

71. Please indicate the percent of personal responsibility that you felt for the outcomes of your patients over the past 2 weeks, regardless of whether the outcomes were positive or negative (0%=you did not feel responsible for patient outcomes; 50% you shared responsibility equally

with other providers or other factors leading to your patient's illness; 100%=you felt fully responsible for patient outcomes)

0 10 20 30 40 50 60 70 80 90 100

---

72. In the past 3 months, have you done any of the following (select all that apply):
    - Taken a vacation (5 or more days off site)
    - Left your site for a weekend or short-term trip (<5 days)
    - Had friends and/or family visit your site
    - Returned to the US to see friends and/or family (previously planned trip)
    - Emergently returned to the US to see friends and/or family (previously unplanned trip, for example for a medical emergency)
    - Met up with friends and/or family somewhere else (not going to the US and not coming to your site)

73. How often do you communicate with family and friends back home (via phone, email, Skype, or social media)?

Never Almost Never Sometimes Fairly Often Very Often

74. This frequency is:

Far Too Little Too Little About Right Too Much Far Too Much

75. How difficult is it to communicate with family and friends back home?

Very difficult Difficult Neutral Easy Very easy

76. In the past 3 months, have you had any of the following occur (select all that apply):
    - Minor issue(s) with your physical or mental health
    - Serious issue(s) with your physical or mental health
    - No issues with physical or mental health

    - Minor safety or security issue(s)
    - Major safety or security issue(s)
    - No safety or security issues

77. Comments about personal or professional factors are impacting your well-being at this point in time. (free-text)

[THIS QUESTION IN FINAL QUARTERLY SURVEY ONLY]

78. Prior to leaving my GHSP site?, I felt overwhelmed by the process of returning home.

Strongly Disagree Disagree Neutral Agree Strongly Agree Strongly Agree

----

The final two questions are about how you feel at this point in time, recognizing and reflecting on your experiences with GHSP to date.

79. Please choose the description that best represents how you feel today:
    - I am excited [everything is new]
    - I am frustrated, irritable, and feel judgmental toward others.
    - I am well able to cope with day-to-day challenges, though at times still feel frustrated.
    - I have incorporated new behaviors that fit with this culture and generally function well with challenges that I encounter.

80. At the moment, I would like to incorporate global health into my career when I finish GHSP.

Strongly Disagree Disagree Neutral Agree Strongly Agree

Unpublished key, not to be included in the survey:

1. *Represents Honeymoon stage of culture shock*
2. *Represents Frustration stage of culture shock*
3. *Represents Adjustment stage of culture shock*
4. *Represents Acceptance stage of culture shock*

at end:

"The data collected as part of this survey will be used anonymously, therefore we will not be able to identify volunteers who may be having a difficult time right now. If you are in need of assistance, please reach out to the appropriate person at Peace Corps or Seed.

POST-SERVICE SURVEY, T_5 to be administered ~3 months after completion of service

Thank you for participating in this project. The data being collected in these surveys is meant to help the GHSP team (Seed and Peace Crops) understand the personal and contextual factors that influence your experience with GHSP, specifically looking at resiliency, level of stress, and the experience of culture shock. This will then be used to identify support methods to promote successful functioning before, during, after service.

Please take 30 minutes to complete this survey. Your MGH ID is being used in order to connect your answers with future survey responses, but will be replaced with a unique study number so no one analyzing the data can connect answers to a specific individual. Additionally, the data collected online will be deleted from the system once each survey round is complete. Thank you for your time!

[CD-RISC 10 Resilience Assessment Tool[20,22,23]*]*
Please indicate how much you agree with the following statements as they apply to you over the last month, using the number scale below. If a particular situation has not occurred recently, answer according to how you think you may have felt.

	0=Not true at all	1=Rarely true	2= Sometimes true	3=Often true
1. I am able to adapt when changes occur				
2. I can deal with whatever comes my way				
3. I try to see the humorous side of things when I am faced with problems				
4. Having to cope with stress can make me stronger				
5. I tend to bounce back after illness, injury, or other hardships				
6. I believe I can achieve my goals, even if there are obstacles				
7. Under pressure, I stay focused and think clearly				
8. I am not easily discouraged by failure				
9. I think of myself as a strong person when dealing with life's challenges and difficulties				
10. I am able to handle unpleasant or painful feelings like sadness fear and anger				

Returning home

	Strongly Disagree	Disagree	Neutral Agree	Strongly Agree	Strongly Agree	N/A
11. Prior to leaving my GHSP site?, I felt overwhelmed by the process of returning home.						
12. Reintegration to my life in the U.S after GHSP was a challenge.						

13. I am fully reintegrated in my home culture.						
14. I feel supported by the GHSP network in dealing with difficult situations upon returning home.						

15. What personal or professional factors impacted your well-being most when you returned home? (free text)

16. What more could be done by GHSP to assist with reintegration after GHSP service/ the GHSP year?

	Strongly Disagree	Disagree	Neutral Agree	Strongly Agree	Strongly Agree	N/ A
17. Reflecting on my work with GHSP, I was able to make a positive difference at my site.						
18. Others have been interested in hearing about my experience with GHSP.						
19. Others in my field have seen value in my experience with GHSP.						
20. Working in global health is a core part of my self-identity.						

21. Have you or do you plan to incorporate these components of global health into your career?

	Very unlikely	Unlikely	Unsure	Somewhat likely	Very likely
International research					
International health advocacy					
International health education (precepting, teaching, mentorship and/or training)					
International clinical work					
Stateside global health education					
Domestic clinical work in low resource settings					
Domestic advocacy work					
Domestic health disparities research					
Domestic health education (precepting, teaching, mentorship and/or training)					

Reflecting on your time with GHSP:

	None	Slight	Moderate	Great
22. Over the course of the year, how much culture shock did you experience on average? Please explain:				

23. Over the course of the year, how stressed did you feel on average??<br><br>Please explain:				

	Decrease d	Stayed the same	Increase d
24. How do you feel GHSP has changed your resilience?			
